# Alteration of gene expression by alcohol exposure at early neurulation

**DOI:** 10.1186/1471-2164-12-124

**Published:** 2011-02-21

**Authors:** Feng C Zhou, Qianqian Zhao, Yunlong Liu, Charles R Goodlett, Tiebing Liang, Jeanette N McClintick, Howard J Edenberg, Lang Li

**Affiliations:** 1 Department of Anatomy and Cell Biology, Indiana University School of Medicine, 635 Barnhill Drive, Indianapolis, IN, 46202, USA; 2 Department of Medicine, Indiana University School of Medicine, 545 Barnhill Drive, Indianapolis, IN, 46202, USA; 3 Department of Biochemistry and Molecular Biology, Indiana University School of Medicine, 635 Barnhill Drive, Indianapolis, IN 46202, USA; 4 Department of Psychology, Indiana University-Purdue University at Indianapolis 402 N. Blackford Street, Indianapolis, IN, 46202, USA

## Abstract

**Background:**

We have previously demonstrated that alcohol exposure at early neurulation induces growth retardation, neural tube abnormalities, and alteration of DNA methylation. To explore the global gene expression changes which may underline these developmental defects, microarray analyses were performed in a whole embryo mouse culture model that allows control over alcohol and embryonic variables.

**Result:**

Alcohol caused teratogenesis in brain, heart, forelimb, and optic vesicle; a subset of the embryos also showed cranial neural tube defects. In microarray analysis (accession number GSM9545), adopting hypothesis-driven Gene Set Enrichment Analysis (GSEA) informatics and intersection analysis of two independent experiments, we found that there was a collective reduction in expression of neural specification genes (neurogenin, *Sox5, Bhlhe22*), neural growth factor genes [*Igf1, Efemp1*, *Klf10 *(*Tieg), and Edil3*], and alteration of genes involved in cell growth, apoptosis, histone variants, eye and heart development. There was also a reduction of retinol binding protein 1 (*Rbp1*), and *de novo *expression of aldehyde dehydrogenase 1B1 (*Aldh1B1*). Remarkably, four key hematopoiesis genes (glycophorin A, adducin 2, beta-2 microglobulin, and ceruloplasmin) were absent after alcohol treatment, and histone variant genes were reduced. The down-regulation of the neurospecification and the neurotrophic genes were further confirmed by quantitative RT-PCR. Furthermore, the gene expression profile demonstrated distinct subgroups which corresponded with two distinct alcohol-related neural tube phenotypes: an open (ALC-NTO) and a closed neural tube (ALC-NTC). Further, the epidermal growth factor signaling pathway and histone variants were specifically altered in ALC-NTO, and a greater number of neurotrophic/growth factor genes were down-regulated in the ALC-NTO than in the ALC-NTC embryos.

**Conclusion:**

This study revealed a set of genes vulnerable to alcohol exposure and genes that were associated with neural tube defects during early neurulation.

## Background

Children born to women who drink heavily during pregnancy are at risk for various developmental disorders, collectively called Fetal Alcohol Spectrum Disorder (FASD). Fetal Alcohol Syndrome (FAS) is a severe form of FASD in which the affected child is diagnosed with growth retardation, abnormal central nervous system development (typically including microencephaly), and a characteristic pattern of abnormal facial features [1-4]; organ dysmorphology, particularly of the eye and heart, may be evident in FAS cases as well [5,6]. Disruption of complex molecular cascades that regulate embryonic morphogenesis likely are responsible for the teratogenic effects of alcohol. Potential mechanisms include metabolic stress, reduced signaling by transcription factors, retinoic acid or growth factors, disrupted cell-cell interactions, impaired cell proliferation, and apoptosis [7-16]. Several of these mechanisms may have direct roles in causing the cell death and growth retardation in multiple systems, including brain and head (for review see [17]).

Expression of a number of genes during development was reported to be affected by alcohol in different experimental paradigms, including homeobox genes such as *Msx2 *[18] and sonic hedgehog [19,20], neurotrophic molecules (e.g. ADNP gene [21]), fetal liver kinase 1 (*Flk1*) [22]), retinol-related genes (e.g. Crabp1 and Fabp4; [20]), nucleotide excision repair gene, (*Ercc6l*) [23], stress-related genes (e.g. heat shock protein 47 [24]), and differentiation and apoptosis genes such as *Timp4, Bmp15, Rnf25, Akt1, Tulp4, Dexras1 *[25]. These altered genes suggest potential mechanisms for the abnormal development in FASD. However, the wide-ranging developmental abnormalities in FASD are likely a consequence of the interaction of multiple genes. Examination of global gene expression provides a holistic view of genes that potentially interact and collaboratively contribute to the abnormal development. Alcohol exposure induced changes in a group of cellular adhesion genes (e.g. *L1cam *and *integrin*) in neuroblastoma cells [26]. A brief ethanol exposure (3 h) at gestation day 8 (E8) in mouse embryos altered expression of genes of metabolic, cell programming and cytoskeletal signaling pathways [27]. An earlier alcohol exposure at E6-E8 also altered a set of genes related to PLUNC, neurofilament, and pale ear [28].

In animal models of prenatal alcohol exposure, sources of variability include the pattern, concentration, amount, and developmental stage of alcohol exposure, maternal stress, embryonic growth and maturation of embryos between litters and even within a given litter and within inbred strains of mice [29]. Control of all these variables in rapidly developing embryos is virtually unattainable *in vivo*. To limit these variables, a whole embryonic culture [30,31] was adopted, including stage alignment based on somite number, in which the pattern, amount and concentration of alcohol and embryonic staging were controlled. Inbred C57BL/6 mice, with known susceptibility to ethanol teratogenesis [32,33], were used for this study.

Differences in the dose and timing of alcohol exposure are known contributors to variation in the phenotypic spectrum in FASD. Understanding the pattern of gene alterations that co-vary with different outcomes produced by different alcohol doses or developmental timing of exposure would provide valuable insights into mechanisms underlying this phenotypic variability. As development is highly dynamic throughout gestation, we asked how alcohol exposure might affect genome-wide gene expression at the critical stage of neurulation (E8-10), when the nervous system (and other major organs) are actively forming in mouse. We have shown that at this key stage, neural tube formation was highly sensitive to the alcohol insult [29]. DNA methylation was altered, with the degree of change commensurate with severity of neural tube defect [34]. In the current study, in an initial experiment, cluster analysis indicated distinct differences in gene expression not only between control- and alcohol-treated embryos, but also between two phenotypic subsets of alcohol-treated embryos discernable at the end of alcohol treatment, one group which had a closed neural tube (ALC-NTC) and the other group with an open neural tube (ALC-NTO). A second study with a larger set of arrays was then performed in which alcohol-treated embryos of both neural tube phenotypes were specifically compared. We report here the correlation of alcohol-induced embryonic growth retardation and neural tube abnormalities with changes in expression in networks of genes known to regulate embryonic growth, organ development, and neural specification processes.

## Results

### Embryonic Growth Retardation/Abnormalities

As was seen in our previous report [29], the size and somite number varied (from 1-6) among embryos within a litter at the time of harvesting from the mother. We selected embryos of similar developmental stages (3-5 somites) and randomly assigned them to the two treatment groups (alcohol or control). The alcohol concentration profile of the culture media over the 46 hours was similar to that in our previous report [29]. The concentration of ethanol in the medium was ~88 mM at the start of each day (when first added to the media) and declined to ~44 mM by the end of each day. Among all cultured embryos, more than 95% maintained active heartbeats and blood circulation over this time, and only those were used for analysis. Development of the heart, caudal neural tube, brain vesicles, optic system, and limb buds in the embryos were significantly compromised in the alcohol treated group (Table 1). Brain vesicle development was retarded and the brain vesicles were smaller in size in the alcohol group. The significant effects in multiple organs and regions and in total scores (Table 1) demonstrated that alcohol treatment resulted in retardation of the overall growth and interfered with development of several specific structures, including brain, heart, and limb development, in this embryonic culture model.

**Table 1:** Embryonic dysmorphology after alcohol exposure, scored according to Maele-Fabry et al,1992.

Region	Control	Alcohol	ALC-NTC	ALC-NTO
Allantois	3 ± 0	2.80 ± 0.08	2.86 ± 0.10	2.70 ± 0.14
Branchial bars	2.77 ± 0.09	2.15 ± 0.21	2.17 ± 0.26	2.11 ± 0.39
Brain: Forebrain	4.76 ± 0.10	3.81 ± 0.27*	4.57 ± 0.14	2.29 ± 0.29** ^^
Brain: Midbrain	4.52 ± 0.11	3.71 ± 0.27	4.50 ± 0.14	2.14 ± 0.14** ^^
Brain: Hindbrain	4.71 ± 0.10	3.86 ± 0.24*	4.50 ± 0.14	2.57 ± 0.30** ^^
Caudal Neural Tube	4.76 ± 0.12	4.11 ± 0.19*	4.09 ± 0.26*	4.14 ± 0.26*
Flexion	4.80 ± 0.09	4.33 ± 0.19	4.59 ± 0.19	3.81 ± 0.36*
Heart	4.80 ± 0.10	4.10 ± 0.16**	4.15 ± 0.19*	4.00 ± 0.31*
Limb: Forelimb	2.01 ± 0.06	1.51 ± 0.13**	1.48 ± 0.18*	1.57 ± 0.20
Limb: Hindlimb	0.53 ± 0.10	0.20 ± 0.08*	0.21 ± 0.09	0.19 ± 0.14
Mandibular process	2.08 ± 0.11	1.99 ± 0.09	2.12 ± 0.08	1.71 ± 0.18
Maxillary process	2.41 ± 0.14	2.06 ± 0.16	2.21 ± 0.18	1.76 ± 0.30
Olfactory system	0.47 ± 0.08	0.26 ± 0.08	0.29 ± 0.11	0.20 ± 0.13
Optic system	3.59 ± 0.14	2.87 ± 0.14**	3.02 ± 0.17*	2.57 ± 0.20**
Otic system	3.95 ± 0.12	3.68 ± 0.11	3.88 ± 0.10	3.29 ± 0.18* ^
Somites	4.81 ± 0.09	4.38 ± 0.16	4.50 ± 0.17	4.14 ± 0.34
Total score	53.97 ± 0.66	45.83 ± 1.54**	49.14 ± 1.54**	39.23 ± 1.72** ^^

* P-Value <0.05, ** <0.01; compared to control

^ P-Value <0.05, ^^ <0.01; compared to NTC

Control n = 21; Alcohol (all alcohol-treated, n = 21; ALC-NTC, n = 14; ALC-NTO, n = 7)

**Figure 1:**
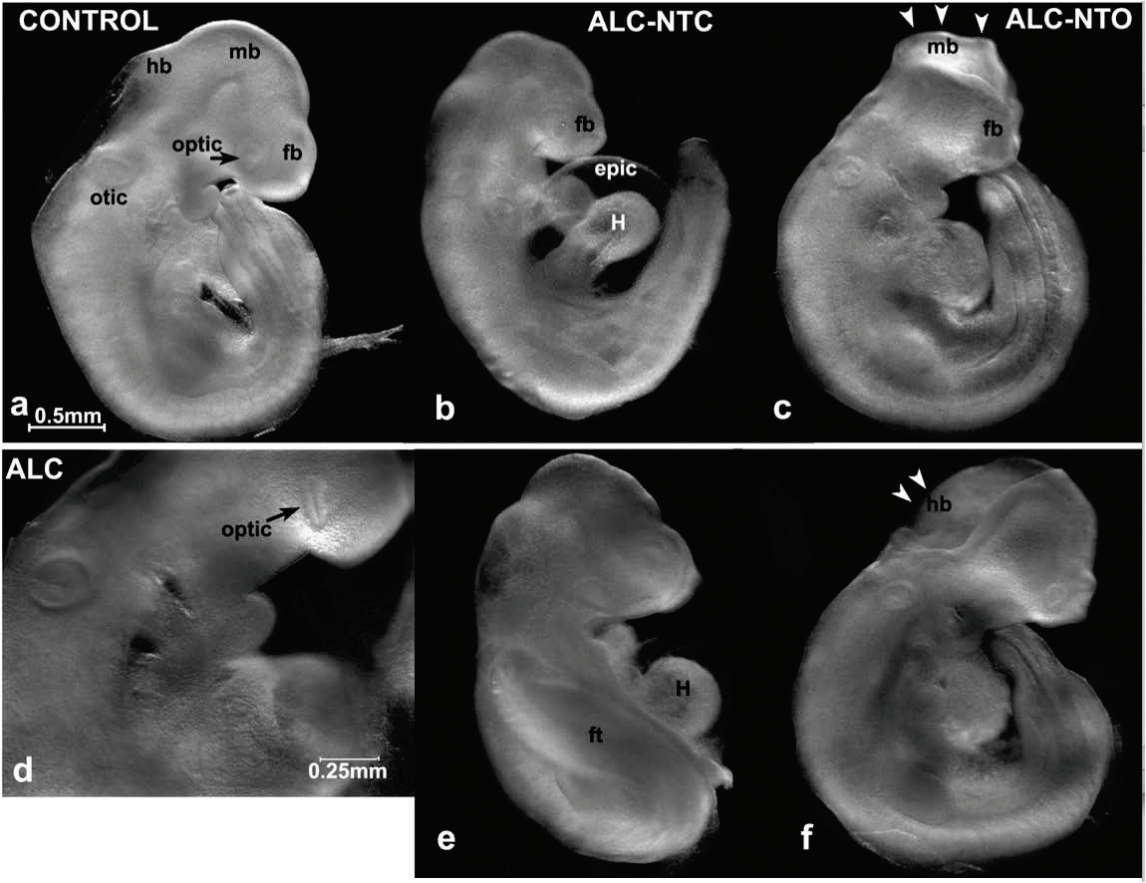
**Alcohol causes dysmorphology of growing embryos**. Control embryos (a), Alcohol-treated (b-f). There are many dysmorphologies including microencephaly of forebrain (b, c, f), failure of closure of midbrain (mb; c) or hindbrain (hb; f), dysmorphic optical vesicle (optic; d), flex tail (ft; e) in caudal neural tube, delay formation of heart (H) chamber (b) and occasional detachment of epicardium (epic; b and e), neural tube opening at midbrain (mb; c, arrowheads) and hindbrain (hb; f, arrowheads) in the alcohol group. Majority of the brain vesicles in alcohol-treated group were closed (ALC-NTC; b, e). Approximately 30% of the embryos were found with a neural tube opening (ALC-NTO), usually in the head fold. Scale bars: a, b, c, e, f = 0.05mm; d = 0.25 mm.

**Figure 2:**
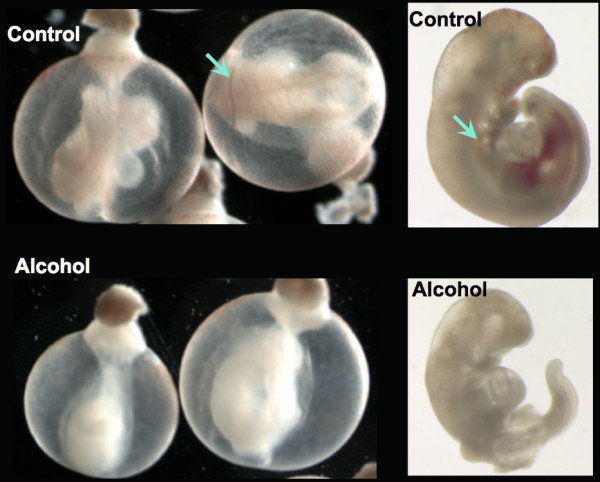
**The red blood vessels were less distinguishable in the yolk sac (arrow, left) and embryo (arrow, right) in the alcohol-treated group as compared with those of the Control**. All embryos examined for red blood vessels had active heart beat at the termination of experiment.

The overall growth retardation was accompanied by varying degrees of abnormality in organ system development (Figure 1). These abnormalities included an increased size of the heart and ventricular chambers, reduced size of lung buds, flattened forebrain, small/slanted eyes, abnormal tail morphology, abnormal limb web, and unfinished turning of neural axis. A reduced blood/vascular system was also evident by less vascularization in yolk sac (Table 1), and lower red coloration apparent in many blood vessels of yolk sacs and embryos in the alcohol-treated than the control embryos (Figure 2).

Among 127 samples of alcohol-treated embryos, 34 (27%) had various degrees of incomplete neural tube closing (Figure 1); this compares to 3 (2%) out of the 139 controls. These openings in the neural tube mostly occurred in the head fold, although delayed or incomplete neural tube closure in midbrain and hindbrain was also seen. The abnormalities and developmental delays are clearly more severe in ALC-NTO than in ALC-NTC subgroups, particularly in development of the neural axis including hindbrain, midbrain, forebrain, otic vesicle.

### Differences in Gene Expression

**Figure 3:**
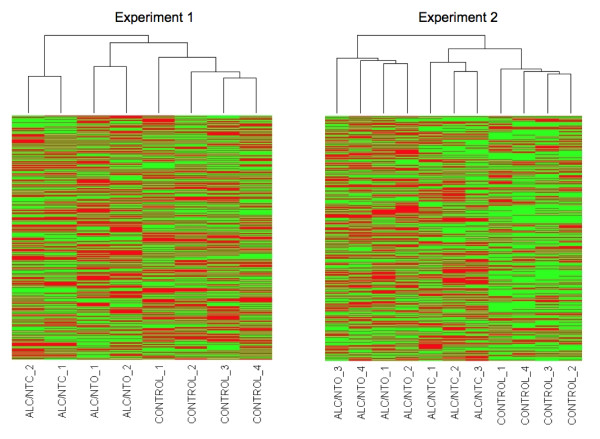
**Hierarchical clustering by arrays in Experiment 1 and Experiment 2**.

At the end of the culture period, the total RNA extracted from alcohol-treated embryos was approximately half that of controls: controls = 2.8 ± 0.5 (n = 13), ALC-NTC = 1.6 ± 0.5 (n = 13, P < 0.05 compared with control), ALC-NTO = 1.2 ± 0.5 (n = 8, P < 0.05 compared with control). In Experiment 1, 14,243 out of 22,690 probe sets (62.7%) were present in at least half of the samples in either control or alcohol treated groups. Hierarchical clustering by arrays (Figure 3. **Exp 1**) clearly separated the samples into three groups, control, ALC/NTC, and ALC/NTO, rather than just two (ALC vs. control). In Experiment 2, 26,674 out of 45101 probe sets (59.1%) were present in at least half of the samples in either control or alcohol treated group. Again, the hierarchical cluster analysis (Figure 3. **Exp 2**) separated the samples into the same three groups, control, ALC/NTC, and ALC/NTO.

In Experiment 1, 850 probe sets (6% of the probe sets that were present) were differentially expressed in alcohol-treated embryos as a group (p ≤ 0.05). In Experiment 2, which had more power due to the larger number of arrays and also examined twice as many probe sets, 2519 probe sets (9.4% of the probe sets that were present) were differentially expressed in alcohol-treated embryos considered as a group (p ≤ 0.05). These relaxed stringencies were employed to reduce false negatives when comparing genes across the two experiments. The probe sets on the Mouse Genome 430A GeneChip were a subset of those on the Mouse Genome 430 2.0 GeneChip. Comparing this common subset across the two experiments, 87 probe sets were significant in both experiments and consistent in direction; because there are 13810 genes present in both experiments, the null expectation is that only 17 genes would be expected to be in common with the same direction of change. 49 probe sets were lower in alcohol-treated embryos and 38 were higher (Table 2). Among these were genes for alcohol metabolism, epigenetics (histone and histone variants), hematopoiesis, neurotrophic factors, retinol metabolism, cell cycle, cell adhesion, homeobox genes, and oncogenes.

**Table 2:** Genes with changed expression^†^

Gene symbol	UniGene	Source.id	Exp. 1 Fold Change	Exp. 2 Fold Change	Category/Function	Description
AI415282	Mm.254704	1415793_at	-1.1	-1.6		expressed sequence AI415282
Atp6ap2	Mm.25148	1439456_x_at	-1.2	-1.2	Energy	ATPase, H+ transporting, lysosomal protein 2
BC008163	Mm.11473	1425328_at	-1.2	-1.2		CDNA sequence BC008163
Cask	Mm.253779	1427692_a_at	-1.5	-1.1	MAGUK family	calcium/calmodulin-dependent serine protein kinase
Clk1	Mm.1761	1426124_a_at	-1.6	-1.3	Cell cycle	CDC-like kinase 1
Clk4	Mm.239354	1427663_a_at	-1.7	-1.3	Cell cycle	CDC like kinase 4
Cri1	Mm.44244	1448406_at	-1.2	-1.2	DNA transcription/differentiation	CREBBP/EP300 inhibitory protein 1
Cyr61	Mm.1231	1416039_x_at	-1.4	-1.6	extracellular matrix	Cysteine rich protein 61
Dach2	Mm.79760	1449823_at	-1.3	-1.3	Myogenin	Dachshund 2 (Drosophila)
Ebf1	Mm.255321	1416302_at	-1.5	-1.6	hematopoiesis	early B-cell factor 1
Ebf2	Mm.319947	1449101_at	-1.2	-1.4	hematopoiesis	early B-cell factor 2
Ebf3	Mm.30282	1428349_s_at	-1.5	-1.3	hematopoiesis	early B-cell factor 3
Edil3	Mm.41716	1433474_at	-1.5	-1.4	Homeobox	EGF-like repeats and discoidin I-like domains 3
Efemp1	Mm.44176	1427183_at	-1.4	-1.9	Neurotrophin	EGF-containing fibulin-like extracellular matrix protein 1
Foxd1	Mm.347441	1418876_at	-1.4	-1.2	Homeobox	forkhead box D1
Gypc	Mm.292145	1423878_at	-1.2	-1.4	hematopoiesis	glycophorin C
Hist1h3a	Mm.221301	1422948_s_at	-1.6	-1.3	Epigenetic	histone 1, H3a
Hist1h4i	Mm.14775	1424854_at	-1.7	-1.5	Epigenetic	Histone 1, H4i
Hist3h2a	Mm.212549	1435866_s_at	-2.1	-1.7	Epigenetic	histone 3, H2a
Igf1	Mm.268521	1419519_at	-1.4	-1.3	Neurotrophin	insulin-like growth factor 1
Lgals1	Mm.43831	1419573_a_at	-1.4	-1.7	Angiogenesis/neural development	Lectin, galactose binding, soluble 1
Mageh1	Mm.6890	1422498_at	-1.3	-1.3	Oncogene	Melanoma antigen, family H, 1
Myct1	Mm.33762	1452072_at	-1.3	-1.3	Oncogene	myc target 1
Napb	Mm.274308	1423172_at	-1.6	-1.4	Synapsis	N-ethylmaleimide sensitive fusion protein beta
Ndrg1	Mm.30837	1423413_at	-1.9	-1.8	Cell cycle	N-myc downstream regulated gene 1
Peli1	Mm.28957	1417371_at	-1.2	-1.1	Kinase	Pellino 1
Pim1	Mm.328931	1435872_at	-1.3	-1.3	hematopoiesis	proviral integration site 1
Ppox	Mm.300006	1416618_at	-1.3	-1.3	hematopoiesis	protoporphyrinogen oxidase
Ppp1r14a	Mm.2343	1418086_at	-1.1	-1.3	signal transduction	Protein phosphatase 1, regulatory subunit 14A
Ptx3	Mm.276776	1418666_at	-1.5	-1.7	plasma proteins	pentaxin related gene
Rab11a	Mm.1387	1449256_a_at	-1.1	-1.2	Oncogene	RAB11a, member RAS oncogene family
Rbp1	Mm.302504	1448754_at	-1.2	-1.2	Retinol metabolism	retinol binding protein 1, cellular
Rpl13a	Mm.180458	1433928_a_at	-1.1	-1.1	Synthesis	ribosomal protein L13a
Rpl17	Mm.276337	1453752_at	-1.3	-1.2	Synthesis	ribosomal protein L17
Skil	Mm.15406	1422054_a_at	-1.6	-1.4	Oncogene	SKI-like
Sncg	Mm.282800	1417788_at	-4.4	-1.5	Oncogene	synuclein, gamma
Stmn2	Mm.29580	1423281_at	-1.7	-1.8	Neural specification	Stathmin-like 2
Stmn3	Mm.2319	1460181_at	-1.7	-1.7	Neural specification	Stathmin-like 3
Syap1	Mm.44207	1416472_at	-1.2	-1.1	Synapsis	Synapse associated protein 1
Timp3	Mm.4871	1419089_at	-1.3	-1.3		Tissue inhibitor of metalloproteinase 3
Ube2b	Mm.280233	1423107_at	-1.1	-1.1	Epigenetic	ubiquitin-conjugating enzyme E2B, RAD6 homology
Vcam1	Mm.76649	1448162_at	-1.3	-1.3	Cell adhesion	Vascular cell adhesion molecule 1
1110008H02Rik	Mm.28311	1436506_a_at	-1.3	-1.3	Energy	RIKEN cDNA 1110008H02 gene
2010011I20Rik	Mm.30013	1424695_at	-1.2	-1.6		RIKEN cDNA 2010011I20 gene
2310034L04Rik	Mm.41891	1426416_a_at	-1.2	-1.2		RIKEN cDNA 2310034L04 gene
5033414D02Rik	Mm.275511	1460361_at	-1.2	-1.3		RIKEN cDNA 5033414D02 gene
5230400G24Rik	Mm.139176	1451572_a_at	-1.3	-1.1		RIKEN cDNA 5230400G24 gene
5730420B22Rik	Mm.28129	1427050_at	-1.4	-1.4		RIKEN cDNA 5730420B22 gene
A630082K20Rik	Mm.293175	1427359_at	-1.7	-1.3		RIKEN cDNA A630082K20 gene
Acsl6	Mm.267478	1451257_at	1.2	1.4	Lipid metabolism	acyl-CoA synthetase long-chain family member 6
Atp1a1	Mm.280103	1451071_a_at	1.2	1.2	Energy	ATPase, Na+/K+ transporting, alpha 1 polypeptide
AW547365	Mm.270088	1433645_at	1.2	1.2	Membrane Transport	expressed sequence AW547365
C78212	Mm.27090	1435369_at	1.2	1.3		Expressed sequence C78212
Cad	Mm.305535	1452830_s_at	1.2	1.3	Amino acid metabolism	carbamoyl-phosphate synthetase 2, aspartate transcarbamylase, and dihydroorotase
Cdv3	Mm.261025	1415704_a_at	1.2	1.2	Lipid metabolism	carnitine deficiency-associated gene expressed in ventricle 3
Clstn1	Mm.38993	1421861_at	1.2	1.2	Cell adhesion	Calsyntenin 1
Cpd	Mm.276736	1434547_at	1.2	1.2	Protease activity	carboxypeptidase D
E130306I01Rik	Mm.277582	1424419_at	1.1	1.3		RIKEN cDNA E130306I01 gene
Emb	Mm.274926	1415856_at	1.3	1.2	Cell adhesion	embigin
Exosc2	Mm.150972	1426630_at	1.1	1.2	RNA degradation	exosome component 2
Hmga2	Mm.157190	1450780_s_at	1.2	1.1		high mobility group AT-hook 2
Hsd11b2	Mm.5079	1416761_at	1.4	1.4	Steroid Metabolism	Hydroxysteroid 11-beta dehydrogenase 2
Ide	Mm.28366	1423120_at	1.2	1.2	Protease activity	Insulin degrading enzyme
Ifrg15	Mm.253335	1418116_at	1.1	1.1		interferon alpha responsive gene
Ipo11	Mm.132208	1428096_at	1.2	1.2	Nuclear Protein Transport	importin 11
Itga6	Mm.225096	1422445_at	1.1	1.2	Cell adhesion	integrin alpha 6
Klf16	Mm.41513	1416350_at	1.3	1.3	Alcohol metabolism	Kruppel-like factor 16
Ndufs1	Mm.290791	1425143_a_at	1.1	1.1	Energy	NADH dehydrogenase (ubiquinone) Fe-S protein 1
Phf13	Mm.25582	1455175_at	1.2	1.1	Alcohol metabolism	PHD finger protein 13
Podxl	Mm.89918	1448688_at	1.3	1.3	hematopoiesis, kinase	Podocalyxin-like
Psmd3	Mm.12194	1448479_at	1.1	1.2	Proteasome	Proteasome (prosome, macropain) 26 S subunit, non-ATPase, 3
Ptcd1	Mm.332840	1454970_at	1.4	1.3		pentatricopeptide repeat domain 1
Rhou	Mm.168257	1449027_at	1.2	1.2	Signal transduction	ras homolog gene family, member U
Rpo1-4	Mm.135581	1417775_at	1.2	1.1	Synthesis	RNA polymerase 1-4
Saa2	Mm.200941	1419075_s_at	1.7	1.7	Lipid metabolism	serum amyloid A 2
Slc27a4	Mm.330113	1424441_at	1.2	1.3	Lipid metabolism	solute carrier family 27 (fatty acid transporter), member 4
Trp53bp1	Mm.215389	1433659_at	1.1	1.2	Cell cycle	transformation related protein 53 binding protein 1
Ttr	Mm.2108	1454608_x_at	2	1.6	Retinol	Transthyretin
Ube2j1	Mm.259095	1417723_at	1.2	1.3	Epigenetic	ubiquitin-conjugating enzyme E2, J1
0610007A15Rik	Mm.28122	1452132_at	1.8	1.5		RIKEN cDNA 0610007A15 gene
1110060D06Rik	Mm.319964	1430291_at	1.3	1.3		Adult male corpora quadrigemina cDNA, RIKEN full-length enriched library, clone:B230210C03 product:u
1300001I01Rik	Mm.214574	1428106_at	1.2	1.2		RIKEN cDNA 1300001I01 gene
1700017B05Rik	Mm.22712	1429758_at	1.3	1.3		RIKEN cDNA 1700017B05 gene
1700054N08Rik	Mm.157746	1451483_s_at	1.4	1.2		RIKEN cDNA 1700054N08 gene
4632417K18Rik	Mm.1643	1422628_at	1.2	1.1		RIKEN cDNA 4632417K18 gene
4930485D02Rik	Mm.293449	1424810_at	1.3	1.3		RIKEN cDNA 4930485D02 gene
5930416I19Rik	Mm.143908	1452313_at	1.2	1.1		RIKEN cDNA 5930416I19 gene

^†^P < 0.05

Furthermore, in Experiment 2 (which had more power to detect differences), a number of genes in addition to the above list were present in the controls but were absent in the alcohol treated samples (Table 3). Notably, glycophorin A (*Gypa*) and beta-2 microglobulin (*B2m*) genes were absent in ALC-NTO, and ceruloplasmin (*Cp*), adducin 2 (*Add2*), B2 m, and ceruloplasmin (*Cp*) genes were absent in ALC-NTC. All of these are critical in hematopoiesis and/or red blood cell function [35-39]. In contrast, the aldehyde dehydrogenase 1 family, B1 (*Aldh1b1*), which catalyzes oxidation of retinaldehyde, was present only in the alcohol-treated embryos with open neural tubes (ALC-NTO) (Table 3 last row). No gene was found to be absent in Control but present in ALC-NTC. Another retinol regulating gene, cellular retinol binding protein 1 (C*rbp1*), was reduced by alcohol exposure (Table 2).

**Table 3:** Genes in Experiment 2 that are turned on or off by alcohol treatment.

Gene Symbol	Genbank	p-value	Change in Alcohol-treated	Description
**Alc-NTC**				
Add2*	NM_013458	0.0196	Off	Adducin 2 (beta)
B2m*	NM_009735	0.0446	Off	Beta-2 microglobulin
*Cfi*	NM_007686	0.0494	Off	Complement component factor i
Cp*	NM_007752	0.0225	Off	Ceruloplasmin
*Fbxo2*	NM_176848	0.0290	Off	F-box only protein 2
*Gch1*	NM_008102	0.0017	Off	GTP cyclohydrolase 1
*Gfi1b*	NM_008114	0.0007	Off	Growth factor independent 1B
*Nppb*	NM_008726	0.0103	Off	Natriuretic peptide precursor type B
*Pitpnm1*	NM_008851	0.0272	Off	Phosphatidylinositol membrane-associated 1
*Ppgb*	NM_008906	0.0044	Off	Protective protein for beta-Galactosidase
*Tacr2*	NM_009314	0.0108	Off	Tachykinin receptor 2
**ALC-NTO**				
*Acbd5*	NM_028793	0.0292	Off	Acyl-Coenzyme A binding domain containing 5
B2m*	NM_009735	0.0446	Off	Beta-2 microglobulin
*Fbxo2*	NM_176848	0.0290	Off	F-box only protein 2
*Frmd3*	NM_172869	0.0004	Off	FERM domain containing 3
Gypa*	NM_010369	0.00001	Off	Glycophorin A
*Mlr1*	BB298201	0.0295	Off	Mblk1-related protein-1
*Ogn*	NM_008760	0.0363	Off	Osteoglycin
*Pdcd4*	BG230003	0.0468	Off	Programmed cell death 4
*Sqstm1*	NM_011018	0.0357	Off	Sequestosome 1
*Aldh1b1*	NM_028270	0.0048	On	Aldehyde dehydrogenase 1 family, member B1

Off = present in ≥ 75% of arrays in control and in no arrays of alcohol-treated samples.

On = not present in any control, present in ≥ 75% of arrays from alcohol-treated.

Only named genes are shown. * = hematopoiesis gene.

P-value: as compared with Control.

### Gene Set Enrichment Analysis (GSEA) Analyses

Four GSEA analyses were conducted within each experiment: control versus all alcohol-treated (ALC), control versus ALC-NTC, control versus ALC-NTO, and ALC-NTC versus ALC-NTO. As 415 GO gene sets and 191 stem cell related gene set were pre-selected, there were totally 4 × (415+191) = 2424 GSEA tests. We found 15 gene sets that were significant at 5% and shared the same enrichment direction in both experiments. By chance, one would expect only 2424 × (0.05 × 0.05 × 0.5) = 3; therefore, the FDR is 3/15 = 20%. The significant gene sets common to the two experiments are outlined below.

#### a. Early Developmental Biology Gene Sets

**Figure 4:**
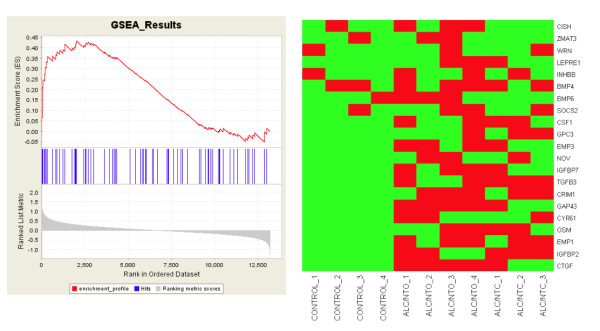
**Illustration of Gene Set Enrichment Analysis (GSEA) informatics with neurotrophic factor related gene set**. (Left panel) Profile of the running enrichment score (ES) and positions of a prominent neurotrophic factor related gene set, GO:0040007: Growth, on the rank ordered list GSEA output for the comparison ALC vs. CONTROL. This test is a one-way test, i.e. whether gene expression is higher in control than in ALC. The x-axis lists all the genes ranked based on their associations with phenotype, i.e. the comparison ALC vs. CONTROL. The blue vertical bars indicate candidate genes in the target gene set. The ES profile records the cumulative score of the gene ranks from the target gene set. If a majority of gene ranks from the candidate gene set are high (i.e. toward the start of ranking) compared to the rest of genes, the cumulative ranking score (profile) will have a high peak, suggesting a significant enrichment of this gene set. The statistical significances (p-value) were calculated based on the height of this peak through a permutation test (p-value = 0.010 in Experiment 1 and 0.005 in Experiment 2). (Right panel) The significant genes (enriched in control) are determined by the position of the peak of the profile. There are 21 candidate genes up to this peak position which are claimed as significant. They are plotted in the Heatmap (green means high expression level, and red means low expression level) in Experiment 2.

GSEA analysis using the GO biological function categories selected as being related to development (Additional files 1 and 2.) identified 20 enriched sets in Experiment 2. Of these 20 sets, 9 were also identified by Experiment 1 (Table 4). Included in these shared gene sets are multiple GO categories related to *growth*, *eye *and *heart development*, and *epigenetics*. When comparing the control embryos to all alcohol treated embryos, there were 7 GO categories that were enriched in the control groups (i.e., down-regulated in the alcohol-treated groups): five growth-related GO sets, one epigenetics (histone and chromatin regulator) GO set, and one angiogenesis GO set (Table 4). No gene set was enriched in the alcohol-treated group. An example of gene enrichment analysis is shown in Figure 4 for GO:0040007, *Growth*. This gene set contained 75 genes. The GSEA p-values for this enrichment score were 0.010 in Experiment 1 and 0.005 in Experiment 2.

**Table 4:** GSEA for *Early Developmental Biology GO sets*.

Comparison	Keyword	Gene Set	Gene Set Description	Size	p-value Exp. 2	Significant Genes	p-value Exp. 1
Control vs ALC-NTO/ALC-NTC (see legends)	**Growth**, Growth Regulation*	*GO:0016049* GO:0040007	***^Cell growth*** **^Growth**	47 75	0.002 0.005	***(Ctgf, Igfbp2, Emp1, Osm, Cyr61, Gap43, Crim1, Tgfb3, Igfbp7, Nov, Emp3), Gpc3, Csf1, Socs2, Bmp6, Bmp4, Inhbb, Lepre1, Wrn, Wig1, Cish***	*0.010* 0.010
		
		*GO:0001558* GO:0040008	***Regulation of cell growth*** **Regulation of growth**	39 56	0.002 0.016	**(*Ctgf, Igfbp2, Osm, Cyr61, Gap43, Crim1, Igfbp7, Nov), Gpc3, Csf1, Socs2*,**	0.006 0.017
		
		GO:0005520	**Insulin-like growth factor binding**	14	0.000	***Ctgf, Igfbp2, Cyr61, Crim1, Igfbp7, Nov***	0.012
	
	Heart*	GO:0001525	**Angiogenesis**	53	0.022	***Ctgf, Anxa2, Cyr61, Thbs1, Vegfa, Tie1, Elk3, Flt1, Crhr2, Vegfc, Kdr, Bmp4, Adra2b, Tnfrsf12a***	0.022
	
	**Eye#**	GO:0001654	**Eye Development**	26	0.040	***Mab21l1, Neurod1, Neurod4, Ntrk2, Fkbp8, Bmpr1b, Crb1, Stat3, Tspan5, Pax6, Bmp4, Map3k1***	0.004
	
	**Epigenetic factor~,^**	GO:0006334	**Nucleosome modeling**	30	0.021	***Hist3h2b, a; Hist1h3f; Hist1h1c; Hist1h2b, c; Hist1h3a; H1f0; Smarca2; Nap1l3***	0.033

*ALC-NTO vs ALC-NTC*	**Growth, Growth retardation**	GO:0007173	**Epidermal growth factor receptor (EGFR) signaling pathway**	5	0.019	***Pde6g, Egfr, Hbegf*** (Enriched in ALC-NTC)	0.023

^= Gene set reduced in ALC-NTO as compared with Control.

*= Gene sets reduced in ALC (all alcohol group) and ALC-NTC as compared with Control.

*~= *Gene set reduced in ALC as compared with Control

**#**= Gene set reduced in ALC-NTC as compared with Control.

**( ) **= genes in Cell growth related GO set, which is included in Growth GO set; or genes in Regulation of Cell growth related GO set, which is included in Regulation of Growth GO set.

The growth-related genes represented the largest group of affected genes. There were 5 GO sets of growth-associated genes (Table 4). Many of these genes, identified by GSEA in both experiments, were also identified in Experiment 2 at the single gene level; e.g. the Growth gene set (GO:0040007): *Ctgf, Igfbp2, Emp1, Osm, Cyr61, Gap43, Crim1, Tgfb3, Nov, Socs2, and Wrn *were significantly reduced in Experiment 2, and *Igfbp7, Emp3, Bmp4, Bmp6, Inhbb, Wig1*, and *Cish *were reduced but did not reach the criteria for significance. The additional growth genes in Epidermal growth factor receptor **(**EGFR) signaling pathway GO group appear to be reduced to a greater extent in ALC-NTO than in ALC-NTC (Table 4).

#### b. Stem Cell Related Gene Sets

Three gene sets were enriched in the control embryos compared to the combined alcohol-treated embryos (i.e., down-regulated in the alcohol-treated group): *TGF-Beta activin-responsive genes *(important for maintenance of pluripotency and embryonic stem cells [40]), *extracellular matrix (ECM) molecules*, and *ECM protease inhibitors ***(**Table 5**)**. Three gene sets were down-regulated in the ALC-NTC subgroup (i.e., enriched in the control group): *other related growth factors *(*Neural specification*), *other regulators of cell differentiation*, and *ECM protease inhibitors ***(**Table 5**)**. Two gene sets were down-regulated in the ALC-NTO group (enriched in the control group): *other related growth factor *and *other ECM molecules *(Table 5). There were no significant gene sets in comparisons between ALC-NTC and ALC-NTO. No gene set was enriched in any alcohol-treated group.

**Table 5:** GSEA for Stem Cell Related Gene Sets

Comparison	Gene Set	Size	p-value Exp. 2	Significant Genes	p-value Exp. 1
*Control vs ALC*	**Other ECM Molecules~^**	9	0.002	***Ctgf, Thbs2, Tgfbi, Ecm1***	0.016
	**ECM Protease Inhibitors~,#**	7	0.004	***Thbs1, Timp3***	0.006
	**TGF-β Activin-responsiv~ e**	16	0.010	***Junb, Fos, Tgfbi, Pdgfb, Tgfb1i1, Igf1***	0.014
	**Other Regulators of Cell Differentiation (Neural Specification) #**	17	0.003	***Elavl3, Neurod1, Neurod4, Nhlh1, Neurog1, Nhlh2, Neurog3, Spock2, Neurog2***	0.004
	**Other Related growth Factor^** **Other Related growth Factor#**	7	0.005 0.040	***Ctgf, Igf1*** ***Ctgf Hgf Igf1***	0.015 0.008

~= Gene set is reduced in ALC (all alcohol group) as compared with Control.

^= Gene set is reduced in ALC-NTO as compared with Control.

#= Gene set is reduced in ALC-NTC as compared with Control.

ECM: extracellular matrix

### Validation by Quantitative RT-PCR

Quantitative RT-PCR (qRT-PCR) was used to verify some of the genes that were significantly affected by alcohol, including a sample of genes from the functional gene sets for neural specification and trophic factors identified in GSEA (Tables 6 and 7). These studies used independent embryos subjected to identical ethanol exposure. The qRT-PCR verified that all 11 down-regulated neural specification genes (Table 6) and neurotrophic/growth factor genes (Table 7) tested differed in the same direction. One gene (*Mylc2*) that did not differ in the microarray experiments was also tested and the lack of difference was confirmed.

**Table 6:** RT-PCR confirmation of differences in gene expression: Neural specification genes from Experiment 1.

		Microarray		qRT-PCR		
Gene	GenBank	Fold Change*	p-value*	Fold Change	p-value	Description
***Bhlhb5***	NM_021560	-1.6	0.0021	-2.3	0	basic helix-loop-helix domain, class B5
***Ngn2***	BC055743	-2	0.0015	-2	0	neurogenin 2
***Ngn1***	NM_010896	-1.3	0.047	-1.7	0.021	neurogenin 1
***Sox5***	AI528773	-1.4	0.015	-1.7	0.005	SRY-box containing gene 5
***Mylc2***	NM_023402	-1	0.8	-1	0.19	myosin light chain

*Control versus alcohol-treated

**Table 7:** RT-PCR confirmation of differences in gene expression: Growth/neurotrophic factor genes from Experiment 2.

		ALC-NTC				ALC-NTO			
		Microarray		qRT-PCR		Microarray		qRT-PCR	
Gene symbol	GenBank	Fold Change*	p-value*	Fold Change*	p-value*	Fold	p-value*	Fold Change*	p-value*
***Ctgf***	NM_010217	-1.7	0.01	-1.2	0.300	-2.2	0.004	-2.0	0.048
***Edil3***	NM146015	-1.6	0.00	-1.4	0.019	-1.9	0.025	Not tested	
***Efemp1***	NM_146015	-1.5	0.01	-2.3	0.002	-2.3	0.001	-1.8	0.010
***Igf1***	NM_184052	-1.4	0.02	-2.6	0.001	-1.3	0.060	Not tested	
***Igfbp2***	NM_008342	-2.0	0.02	-1.6	0.084	-1.5	0.035	-2.0	0.045
***Ntf3***	NM_008742	-1.2	0.30	-1.7	0.060	-1.8	0.030	-1.9	0.038
***Tieg1***	NM_013692	-1.2	0.36	-1.3	0.015	-1.5	0.010	-1.2	0.053

*Control versus alcohol-treated

Fold change: positive = ratio of alcohol-treated to control, negative = ratio of control to alcohol-treated. p-value: t-test of control vs. alcohol treated

## Discussion

### 1. Developmental Deficits and Correlation with Gene Expression Profiles

The abnormal embryonic development resulting from the alcohol treatment at this specific stage of development (Figure 1; Table 1) was consistent with our previous report [29] and those of others [41,42]. Two different facets of abnormal development could be identified: growth delay and frank teratogenesis. Delays in growth were also evident by the significant reductions in the total RNA per embryo and in the delayed morphological staging (Table 1). The affected structures were derived from each of the three germ layers, i.e., neural tube and brain vesicles (ectoderm), somites and cardiovascular system (mesoderm/endoderm), and involved a wide range of tissues and organs (e.g., heart, head, limbs). Alterations in all of these have been observed in FAS cases. The teratogenic consequences were evident as dysmorphology of various organs (central nervous system, eye, and heart) that involved pathogenic effects beyond just the observed delay of the normal course of development. Examples include enlarged heart primordium and abnormally enlarged ventricular chambers, detached pericardial sac, small forebrain, flat telencephalic vesicle, failure in neural tube closure, and small and irregularly shaped eyes.

#### Neural tube defect

We observed in Experiment 1 that gene expression profiles from alcohol treatment of embryos in this controlled culture system yielded two distinguishable patterns; comparison to the morphological data revealed that these were correlated with two different phenotypes: open (ALC-NTO) and closed neural tubes (ALC-NTC). The phenotypes and correlated gene expression differences were reproduced in Experiment 2. The embryos with open neural tubes (ALC-NTO) had more severe delays in brain and otic development than those with closed neural tubes (ALC-NTC) (Table 1). These different phenotypes are consistent with our previous *in vivo *observation in a liquid diet model of prenatal alcohol exposure in C57BL/6 mice, which resulted in partial penetration of incomplete neural tube closure (as late as embryonic day 15) and a cascade of deficits in midline structural development [43]. Finding this difference in development in experimentally controlled culture conditions indicates either a stochastic event or that an extremely sensitive gene-environment interaction is involved, e.g. different outcomes based on small differences in developmental stage at the time of exposure or small differences in tissue concentrations of alcohol across embryos. We have recently found greater DNA hypermethylation in ALC-NTO than in ALC-NTC embryos, particularly in genes on chromosomes 7, 10, and X. Remarkably, there was a >10 fold increase in the number of hypermethlyated genes on chromosomes 10 and X in ALC-NTO than ALC-NTC [34].

Both the ALC-NTC and the ALC-NTO embryos demonstrated lower expression of genes in sets related to cell growth, growth factors, heart (angiogenesis), and eye (in NTC vs. Control) (Table 4**; **Table 7). The ALC-NTC and ALC-NTO embryos also differed in other sets of functionally related genes. The histone gene set was selectively reduced in ALC-NTO compared to controls. The epidermal growth factor signaling pathway genes were lower in ALC-NTO than ALC-NTC (Table 4). At the single gene analysis level, Experiment 2 showed a greater number of neurotrophic/growth factor genes were down-regulated in ALC-NTO than in ALC-NTC groups, particularly in the TGFβ, NTF3, S100, and EGF families. These differences in gene expression between the ALC-NTO and ALC-NTC embryos appear to be correlated with the more severe teratogenic trajectory of the ALC-NTO group, but causal relationships have yet to be established.

The neural tube abnormality may either be a delay in neural tube closure or a neural tube defect. In either case, a delay in closing of the neural tube is associated with deficits in midline brain development due to disruption of the timing of critical events of early brain development. At more mature stages, such midline deficits include craniofacial abnormalities, corpus callosum, olfactory bulb, cerebellum, and raphe neuron formation [43-50].

### 2. Patterns of Gene Expression

#### A. Temporal patterns

Green and colleagues [27] reported that a 3 to 4 h binge-like alcohol exposure, with blood alcohol concentration 300 to 400 mg/dL at E8, produced a major abnormality in craniofacial and eye development in C57BL/6 mice at E15 or E17 (effects in the C57BL/6J substrain were greater than in the C57BL/6N substrain). Alterations of gene expression were reported to occur within hours of alcohol exposure at E8; these genes included metabolic and cellular gene, down-regulated ribosome and proteasome pathways; upregulated glycolysis and pentose phosphate, tight junction, and Wnt signaling pathways, as well as other cellular profile genes. In another study, a comparable high dose of alcohol exposure at an earlier stage, E6-E8, produced growth retardation, abnormal tail torsion, open neural tube, reduction of somite number, and other malformations [28]. The altered gene expression at E10 included cytoskeletal (Neurofilament), signal transduction (Zinc finger protein, MAP kinase related, Transcription factor Nf2l2), and metabolic genes (lactate dehydrogenase, Aldolase 1). In the current study, a similar dose of alcohol exposure at the stage of neurulation (E8-10) produced a major neural and cardiovascular retardation and other organ system abnormalities. The trends of gene expression are consistent with the observed developmental delay and growth retardation in FASD. Among the genes with reduced expression in the alcohol-treated embryos were those involved in growth retardation, neural development, heart and hematopoiesis, and epigenetics. Among the identified functionally related gene sets, the most notable effect was the down regulation of growth-related genes, which represented the largest group of affected genes (Table 4). These genes provide plausible candidates for mechanistic links to the observed embryonic growth retardation.

#### B. Neural specification genes

Expression of neural specification genes (Table 5 and 7) and neurotrophic/growth factor genes (Table 4 and 7) was also reduced by the ethanol exposure. These participate in neuronal specification, neural stem cell differentiation, and neural fate determination [51-55]. Suppression of these genes predicts a downstream reduction in the early formation of neural cells. Null neurog 1 (*Ngn1*) or neurog 2 (*Ngn2*) leads to sensory abnormality [56,57]). These differential expression of neuronal specification/patterning genes together with neurotrophic genes supports the dysmorphism and developmental delay of neural tube and fore-to mid-brain formation. The *Igf1 *and EGF genes were also identified by a microarray study with 3 h alcohol treatment [27] indicating they are altered early after ethanol exposure. The down-regulation of these neural specification and neural trophic/growth factor genes may play a major role in the neurodevelopmental deficit observed in the current study and featured in FASD.

#### C. Genes related to other organ defects

Although heterogeneity of tissue arising from use of whole embryos might have masked some changes in specific tissues, two functional gene sets, optic vesicle and the heart (Table 4), were identified and specifically linked to our observed developmental delay and abnormalities. Also, the collective down-regulation of key hematopoiesis genes that were either absent (Table 3) or reduced (Table 2) is consistent with the reduced blood circulation observed in the embryos.

#### D. Histone variants

Many histone genes related to epigenetic regulation of transcription were affected by ethanol (Table 4). The reduction of many histone variants would alter chromatin organization, affecting transcription at a global level [58,59]; this may be an important effect of the alcohol that leads to the reduction of total RNA and induced growth retardation. Modification of epigenetic processes is a potential mechanism by which alcohol may alter gene expression during development, and may be an important candidate mechanism for the pathophysiology of fetal alcohol syndrome.

#### E. Alcohol delayed or induced gene expression

Other genes that were present in the control group but absent in the alcohol-treated group (Table 3) likely reflect a delay in onset or a strong inhibition of normal expression at this stage of development. Among them, four hematopoiesis genes [glycophorin A (*Gypa*), adducin 2 (*Add2*), beta-2 microglobulin (*B2m*), and ceruloplasmin (*Cp*)] associated with blood cell formation were absent in the alcohol-treated groups; these genes are key components in the pathway of white and red blood cell formation [36,38,60-62]. The absence of these genes is in agreement with the low circulating blood cells seen in alcohol treated embryos (Figure 2). The expression of aldehyde dehydrogenase 1B1 (*Aldh1b1*) was induced in both of our experiments by alcohol treatment during this period of early neurulation (Table 2 last row). Because *Aldh1b1 *encodes an efficient enzyme for breakdown of acetaldehyde formed during metabolism of ethanol, this up-regulation is likely a detoxification response to the high level of ethanol in the environment. However, the metabolism of other substrates of this enzyme (e.g., retinoic acid, corticosteroids, biogenic amines, neurotransmitters, and lipids) that are required for normal development may be adversely affected by this increase in *Aldh1b1 *expression [63,64].

## Conclusion

In summary, alcohol exposure during the period of early neurulation at ~E8-E10, is predominantly inhibitory to gene expression, particularly the neural developmental genes. We found major reductions in gene sets involved in neurospecification, neural growth factors, cell growth and hematopoiesis. These effects on gene expression parallel the growth delay and developmental abnormalities including brain, neural tube, eye, heart, blood cells, and embryonic vascularization which are major targets in FASD. Our study, in conjunction with others that use different developmental periods of alcohol exposure, provides an important portfolio of alcohol-induced changes in gene expression associated with altered development. Together, these gene profiles should contribute to the generation of testable new hypotheses concerning the mechanistic path from gene expression changes to embryonic structural deficits, and for causal mechanisms of alcohol-induced teratogenesis (e.g., brain growth retardation, neural tube midline deficit, craniofacial dysmorphology) in fetal alcohol spectrum disorder. Two such hypotheses emerge from the current study. The first is that alcohol causes a delay in development of the nervous system by inhibiting specific sets of genes involved in neural development (*Ngn*, *Nhlh*, *Sox*, *Igf*, *Ntf*, and *Egf*). The second is that neural tube defects are mediated by the inhibition of genes in the epidermal growth factor signaling pathway and genes encoding histone variants.

## Methods

### Embryonic Culture

All experimental procedures were approved by the Institutional Animal Care and Use Committee of the Indiana University School of Medicine (Indianapolis, IN) and are in accordance with the guidelines of the Institutional Animal Care and Use Committee of the National Institute on Drug Abuse, National Institutes of Health, and the Guide for the Care and Use of Laboratory Animals [65]. Two-month-old C57BL/6 mice (~20 g) were purchased from Harlan, Inc. (Indianapolis, IN). Upon arrival, breeder mice were individually housed and acclimated for at least one week before mating began. The mice were maintained on a reverse 12 h light-dark cycle (lights on: 19:00 - 07:00) and provided with laboratory chow and water ad libitum. Two females were placed with one male for two hours between 08:00 and 10:00. When a vaginal plug was detected after the mating period, it was designated as embryonic day 0 (E0). On E8.25 at 15:00, dams were sacrificed using CO_2 _gas. The embryos were treated at this stage, which is the beginning of neurulation. The window of 46 hrs treatment covered the stages of the formation of the major organs, neural specification and patterning. These stages are known to be vulnerable to alcohol [66].

The technique for whole embryo culture was based on the methods described by New [31]. The gravid uterus was removed and placed in sterile PBS (0.1 M phosphate buffer containing saline) at 37°C. The embryo in the visceral yolk sac along with a small piece of the ectoplacental cone (hereafter called embryo, unless otherwise stated) was carefully removed from the deciduas tissues and the Reichert's membrane in PBS containing 4% fetal bovine serum (Sigma, St Louise. MO). After removal, three embryos bearing 3-5 somites (E8.25) were incubated in a culture bottle in 20 mL of medium which consisted of 70% immediately centrifuged heat-inactivated rat serum (Harlan Sprague-Dawley, Inc, Indianapolis, IN) and 30% phosphate buffered saline (137 mM NaCl, 2.7 mM KCl, 0.5 mM MgCl_2_, 8 mM Na_2_HPO_4_, 1.47 mM KH_2_PO_4_, 0.9 mM CaCl_2_, 5.6 mM glucose, 0.33 mM sodium pyruvate, pH7.4) supplemented with 20 units/ml penicillin and 20 units/ml streptomycin (Sigma, St. Louis, MO), and gassed with 5% O_2_, 5% CO_2_, and 90% N_2 _in a rotating culture system (B.T.C. Precision Incubator Unit, B.T.C. engineering, Cambridge, England, 36 rpm) for 2 h. After 2 h, treatment was initiated by transferring embryos into the same medium with or without 88 mM ethanol in isotonic buffer. The bottles were gassed for an additional 20 h with 5% O_2_, 5% CO_2_, and 90% N_2_, and then between 22 h and 46 h with 20% O_2_, 5% CO_2_, and 75% N_2_. The culture medium in alcohol and control cultures was replaced with fresh medium (with or without ethanol, respectively) 22 h after the start of the treatment. In this culture system, it was previously determined that the media alcohol concentration declined from 88 mM to 44 mM over the course of the experiment. Alcohol concentrations in this range (44-88 mM) have been commonly used in whole embryo cultures to generate FAS-related structural malformations [41,42,67] in multiple strains of mice [29], and are comparable to blood alcohol concentrations produced by *in vivo *doses of acute ethanol injections that produce teratogenic effects in mice during this embryonic period [68]. This level, though high, is within the range attained by human alcoholics [69,70].

All cultures were terminated 46 hrs from the beginning of treatment. The concentration of ethanol in the medium was assayed at three time points on each day (0 [initial], 12, and 22 hours on the first day; at 0 [after media change], 12, and 24 hours on the second day) in a separate group of embryos not used for the analyses, to avoid the potential confounding effects of drawing samples from the cultures. Media samples from alcohol- or vehicle-treated cultures were assayed in duplicate for alcohol concentrations using an Analox alcohol analyzer (Analox Instruments USA, Lunenburg, MA).

At the end of culture, viability was confirmed by observing the blood circulation of the yolk sac and the beating heart. Cultured embryos were quickly immersed in 0.7 ml TRIzol (Invitrogen, Carlsbad, CA) and homogenized for extracting total RNA for the RT-PCR and microarray processes (see microarray section, below), or fixed in 4% paraformaldehyde in PBS for the evaluation of the developmental status.

Whole embryos were used because the dysmorphology is observed throughout tissue derived from the three germ layers and in various developing organs (e.g., head fold, caudal neural tube, heart, lung bud, somites, and limbs). Also, dissection of the millimeter size embryos would unavoidably introduce another source of variability: whole embryos yield sufficient total RNA for single embryo analysis, whereas dissected tissues yield too little RNA and would require pooling or amplification for microarray analysis. Although this limits the resolution of genes contributing to different topographic changes, we thought that obtaining a complete gene expression profile in parallel with this widespread alcohol-induced teratogenesis in the embryo would be informative.

### Embryonic dysmorphology

The analysis of embryo dysmorphology was performed as described by van Maele-Fabry et al. [71] and in our previous report [29]. The morphological features of the developing embryo, including the allantois, flexion, heart, caudal neural tube, hind-brain, midbrain, forebrain, otic system, optic system, branchial bars, maxillary process, mandibular process, forelimb, hindlimb, and somites, were examined and scored for any malformations using the ordinal scales of our previous report [29]. Scores for each of the above features were typically not normally distributed, so they were analyzed statistically by the non-parametric Mann-Whitney U test. The number of somites was normally distributed, so those data were analyzed by Student's t-test, using StatView software (SAS Institute, Inc. Cary, NC).

### Gene expression analyses

Two microarray experiments were performed. In Experiment 1, total RNA was isolated from individual whole embryos (4 vehicle control, 4 alcohol treated). Each embryo was immediately immersed in 700 ml TRIzol (Invitrogen) and homogenized using a Polytron. Extraction followed the TRIzol protocol. Ethanol precipitated RNA was resuspended in DEPC water. RNA was cleaned up using RNeasy mini-kit (Qiagen, Valencia, CA) The quality of RNA was assessed by the Agilent Bioanalyzer (Agilent Technologies, Waldbronn, Germany)and by spectrophotometry from 220 nm to 350 nm; concentration was determined from A260. Typical total RNA yields were 5-10 μg/embryo. Microarray analysis was performed at the Center for Medical Genomics at the Indiana University School of Medicine. Labeling and hybridization to Affymetrix Mouse Genome 430A GeneChips^® ^(Affymetrix, Santa Clara, CA) were carried out following the manufacturer's suggested procedure. Fragmented biotinylated RNA from each embryo was separately hybridized to its own GeneChip for 17 hours at 42°C. The microarray analysis revealed striking differences among the 4 alcohol treated samples, which segregated as two separate pairs rather than one set of four; subsequently, it was noted that one pair of embryos had an open neural tube (ALC-NTO) and the other pair had the neural tube closed (ALC-NTC). All 4 control embryos had closed neural tubes.

Experiment 2 was designed to follow-up these initial results and provide an independent test of the gene expression correlations with the two neural tube phenotypes. Total RNA was isolated from individual embryos (4 vehicle control, 7 alcohol treated: 4 ALC-NTO, 3 ALC-NTC). RNA extraction and microarray analysis was as described above, except that Affymetrix Mouse Genome 430 2.0 GeneChips^® ^(Affymetrix, Santa Clara, CA) were used.

The Mouse Genome 430A chip contains over 22,600 probe sets representing transcripts and variants from over 14,000 well-characterized mouse genes. The newer Mouse Genome 430 2.0 Array contains all of the probe sets present on the earlier 430A chip plus additional probe sets for a total of approximately 45,000 probe sets that analyze the expression of over 39,000 transcripts and variants from over 34,000 well characterized mouse genes. The differences in feature size and probe set content make direct comparisons inappropriate, due to scanning and scaling issues, but because the probe sets on the 430A are present on the 430 2.0 array, those can be compared at the level of gene lists.

The data from independent arrays (each with RNA from a single embryo) for each of the treatments were extracted using the Affymetrix Microarray Suite 5.0 (MAS5) algorithm. Data for both experiments have been deposited in GEO/NCBI and have been assigned series accession number GSE9545 and sample numbers GSM241642 through GSM241660.

To minimize false positive results, only genes detected ("present" by the MAS5 algorithm) on at least half of all individual arrays in at least one experimental condition were retained for further analysis. This avoids data that primarily represent "noise" [72,73].

To detect differentially expressed genes, control samples were compared to ALC-NTC samples, or ALC-NTO samples, or their combination, using a Welch's t-test on the log-transformed signals. To see genes that were similarly affected in both experiments, we intersected the gene lists. To avoid missing genes that met a stringent significance threshold in one experiment but were just beyond that threshold in the second, we chose p ≤ 0.05 as the threshold for each experiment. Given that the two experiments were independent, the probability that a gene overlaps by chance and differs in the same up/down direction in both experiments is (0.05)*(0.05)/2 = 0.00125. False discovery rate (FDR) was calculated based on the number of genes expected to be significant and in the same direction in both experiments under the null hypothesis/the number of such genes actually found.

**Hierarchical clustering **with average linkage function was used to construct a dendrogram based upon all genes that were present on at least half of the arrays in an experimental group.

**Gene Set Enrichment Analysis **(GSEA) [74,75] was carried out to identify groups of related genes that were differentially expressed. GSEA analyses were conducted for 4 different comparisons: control vs. ALC, control vs. ALC/NTC, control vs. ALC/NTO, and ALC/NTC vs. ALC/NTO. The top ranked genes in a significant gene set, in the region up to the maximum score, were considered significant. To reduce multiple testing issues, the GSEA in this study was conducted using two gene set databases designed to test the hypotheses that groups of genes related to *Early Development *or *Stem Cells *were differentially affected by alcohol.

(a) *Early Developmental Biology Gene Sets *(Additional file 1): 415 GO categories that were defined by 29 key words were selected (identified gene sets, Additional file 2).

(b) *Stem Cell Related Gene Sets*: 191 GO categories related to stem cells, neurogenesis, osteogenesis, extracellular matrix, developmental signal transduction pathway, cell cycle, growth factor, TGFβ/BMP signaling, Wnt signaling, and notch signaling were developed by Superarray Bioscience http://www.superarray.com. The gene set information is listed in Additional file 3 (shown with consent of Superarray Bioscience, Frederick, MD).

### Quantitative Real-Time Polymerase Chain Reaction (qRT-PCR)

A number of differentially expressed genes detected in Experiment 1 were selected for qRT-PCR validation based on their biological significance. To test selected genes from the neural specification gene group, the total RNA of each embryo was isolated using the RNeasy mini kit (Qiagen, Valencia, CA) as described above. Vector NTI Advance 9.0 software (Invitrogen, Frederick, MD) was used to design the primers for qRT-PCR (Table 8); if possible, at least one primer in each pair spanned an exon-intron boundary. The number of embryos used in the control group varied from 7 to 9 for different genes, and the number used in the alcohol treated group varied from 9 to 11. The cDNA templates were generated from 50 ng total RNA (TaqMan Reverse Transcription Reagents, Applied Biosystems, Foster City, CA) from each individual embryo, and added to PCR reactions that contained 0.1 μM of forward and reverse primers and SYBR Green PCR Master Mix (Applied Biosystems). Triplicate qRT-PCR were performed for each sample in at least 3 experiments (n = 9). The cycle threshold (Ct) for each cDNA template was determined on the ABI Prism 7700 Sequence Detection System. The Ct refers to the cycle number at which the fluorescence of the amplified product reached an arbitrary threshold that was within the exponential phase of amplification. To correct for sample-to-sample variation, *Gapdh *served as an internal reference. Relative values of expression of neural specific genes were determined for each sample using the ΔΔCt method [76], and these values were normalized to the Ct values of *Gapdh*. The average Gapdh Ct values for alcohol treatment and control were the same in each tested sample, making it an appropriate control gene to normalize the expression of the candidate genes of interest.

**Table 8:** Primers for qRT-PCR

Primer	Sequence	RefSeq ID
Bhlhb5-f	CCTATTCAACAGCGTCTCGTCC	NM_021560
Bhlhb5-r	GCTTCTCACTTTCCTCTAGCTTTGG	
Ctgf-f	AGATTGGAGTGTGCACTGCCAAAG	NM_010217
Ctgf-r	TCCAGGCAAGTGCATTGGTATTTG	
Dll 1-f	ATAGCGACTGAGGTGTAAGATGGAAGC	NM_007865
Dll 1-r	CTTCGCCTGAACCTGGTTCTCAG	
Efemp1-f	TCTACCTACGACAAACAAGCCCTGTG	NM_146015
Efemp1-r	AGAGCTTGTGCGGAAGGTTCCTATAC	
Gapdh-f	TCCTGGTATGACAATGAATACGGC	NM_008084
Gapdh-r	TCTTGCTCAGTGTCCTTGCTGG	
Igf1-f	ACTGACATGCCCAAGACTCAGAAGTC	NM_184052
Igf1-r	TGCCTCCGTTACCTCCTCCTGTTC	
Igfbp2-f	CACAGCAGGTTGCAGACAGTGATG	NM_008342
Igfbp2-r	CAGCTCCTTCATGCCTGACTTGAG	
Ntf3-f	TGGTTACTTCTGCCACGATCTTACAGG	NM_008742
Ntf3-r	CTCCTTTGATCCATGCTGTTGCC	
Mylc2a-f	GGAAGAGTTCAAGCAGCTTCTC	NM_023402
Mylc2a-r	ACTTGTAGTCAATGTTGCCGGC	
Neurog1-f	TCCCTCGGCTTCAGAAGACTTCAC	NM_010896
Neurog1-r	AGGCCTAGTGGTATGGGATGAAACAG	
Neurog2-f	GCGTAGGATGTTCGTCAAATCTG	BC055743
Neurog2-r	TCCGCGCTGGAGGACATC	
Sox5 f	AATATGAGTGGAGATTCTGACGGAAGC	AI528773
Sox5	GGCATTCATTGGACGCTTTATGTG	
Tieg1-f	CAGTCCCAGCATTTTGTTTAACGC	NM_013692
Tieg1-r	GCAGCATCGGAGAAAGATTTGAAG	
Edil3-f	GCTCTCAGGCTGTTCAGAACCTTTG	AF031524
Edil3-r	GGCTTTCCTTGGTTCCCAAGTAAAC	

Primers are named according to the gene with-f for forward primer and -r for reverse primer.

After Experiment 2, we decided to test the three groups (control, ALC/NTO, ALC/NTC) as pools, and chose growth/neurotrophic genes. A separate experiment was carried out with embryonic treatments identical to those used in Experiment 1. Whole embryos were homogenized in TRIzol (Invitrogen) using a Mini-Bead-Beater-8 (Bipspec products, INC, Bartlesville, OK), and total RNA isolation was as described above. Two different pools were created for each condition: Control1 (n = 12), ALC/NTC1 (n = 16), ALC/NTO1 (n = 5), Control2 (n = 5), ALC/NTC2 (n = 9), ALC/NTO2 (n = 6). The relative quantification of expression of each RNA pool was performed using the ABI Prism 7700 Sequence Detection System and calculated using the standard curve method (Applied Biosystems, User Bulletin #2; http:////www.appliedbiosystems.com). In each experiment, a relative expression level was determined for the two pools from each group in triplicate; 3-4 repeat experiments were performed, resulting in 18-24 values from each group. The treatment groups were compared with one way ANOVA followed by Student's t test.

## Authors' contributions

FCZ involved in the overall design and coordination of the study. CG involved in the design of the experiment. QZ and LL carried out the statistical analysis, and YL the informatics. TL performed RTPCR analysis. HJE and JNM were involved in experimental design and carried out the microarray study. All authors participated in the writing of the manuscript. All authors read and approved the manuscript.

## Supplementary Material

Additional file 1 **Keyword and GO categories**. List of keywords used and the GO categories identified by these key words for Early Development. Click here for file

Additional file 2 **GO gene sets selected by keywords**. List of gene sets used in GSEA analyses, derived from GO categories selected by key words for Early Development. Title of each set followed by Entrez Gene Ids of the genes in the set. Click here for file

Additional file 3 **GO categories related to Stem Cells**. Gene sets used in GSEA analysis based on GO categories related to Stem Cells. Title of each set followed by Entrez Gene Ids of the genes in the set. Click here for file
